# Efficacy and safety of deferasirox at low and high iron burdens: results from the EPIC magnetic resonance imaging substudy

**DOI:** 10.1007/s00277-012-1588-x

**Published:** 2012-10-21

**Authors:** J. B. Porter, M. S. Elalfy, A. T. Taher, Y. Aydinok, L. L. Chan, S.-H. Lee, P. Sutcharitchan, D. Habr, N. Martin, A. El-Beshlawy

**Affiliations:** 1 UCL Cancer Institute, Department of Haematology, University College London, Paul O’Gorman Building, 72 Huntley Street, London, WC1E 6BT UK; 2 Ain Shams University, Cairo, Egypt; 3 American University of Beirut, Beirut, Lebanon; 4 Medical Faculty, Ege University, Izmir, Turkey; 5 University Malaya Medical Centre, Kuala Lumpur, Malaysia; 6 Institute of Medical and Veterinary Science, Adelaide, Australia; 7 Chulalongkorn University and King Chulalongkorn Memorial Hospital, Bangkok, Thailand; 8 Novartis Pharmaceuticals, East Hanover, NJ USA; 9 Novartis Pharma AG, Basel, Switzerland; 10 Cairo University, Cairo, Egypt

**Keywords:** Iron overload, Iron chelation therapy, Deferasirox, Liver iron concentration

## Abstract

The effect of deferasirox dosing tailored for iron burden and iron loading based on liver iron concentration (LIC) was assessed over 1 year in less versus more heavily iron-overloaded patients in a substudy of the Evaluation of Patients’ Iron Chelation with Exjade®. Deferasirox starting dose was 10–30 mg/kg/day, depending on blood transfusion frequency, with recommended dose adjustments every 3 months. Therapeutic goals were LIC maintenance or reduction in patients with baseline LIC <7 or ≥7 mg Fe/g dry weight (dw), respectively. Changes in LIC (R2-magnetic resonance imaging) and serum ferritin after 1 year were assessed. Adverse events (AEs) and laboratory parameters were monitored throughout. Of 374 patients, 71 and 303 had baseline LIC <7 and ≥7 mg Fe/g dw, respectively; mean deferasirox doses were 20.7 and 27.1 mg/kg/day (overall average time to dose increase, 24 weeks). At 1 year, mean LIC and median serum ferritin levels were maintained in the low-iron cohort (−0.02 ± 2.4 mg Fe/g dw, −57 ng/mL; *P* = not significant) and significantly decreased in the high-iron cohort (−6.1 ± 9.1 mg Fe/g dw, −830 ng/mL; *P* < 0.0001). Drug-related gastrointestinal AEs, mostly mild to moderate, were more frequently reported in the <7 versus ≥7 mg Fe/g dw cohort (39.4 versus 20.8 %; *P* = 0.001) and were not confounded by diagnosis, dosing, ethnicity, or hepatitis B and/or C history. Reported serum creatinine increases did not increase in low- versus high-iron cohort patients. Deferasirox doses of 20 mg/kg/day maintained LIC <7 mg Fe/g dw and doses of 30 mg/kg/day were required for net iron reduction in the high-iron cohort, with clinically manageable safety profiles. The higher incidence of gastrointestinal AEs at lower iron burdens requires further investigation.

## Introduction

Patients with conditions resulting in iron overload, where their liver iron concentration (LIC) exceeds 7 mg Fe/g dry weight (dw), are at an increased risk of progressive organ dysfunction and/or early death due to iron-related complications [1–8]. Transfusion therapy in the treatment of patients with blood disorders such as β-thalassemia major, sickle cell disease (SCD), and myelodysplastic syndromes (MDS) rapidly leads to iron overload and subsequent tissue and organ damage unless effective chelation therapy is used to control tissue iron levels [9, 10]. Key goals in the management of iron overload are, therefore, to decrease LIC below 7 mg Fe/g dw and to maintain LIC below this level over the long term.

Although many studies have shown the efficacy and safety of the oral iron chelator deferasirox in the treatment of iron overload [11–14], there are limited efficacy and safety data in patients with LIC <7 mg Fe/g dw receiving doses of deferasirox at the recommended starting dose of 20 mg/kg/day [15, 16]. The international, multicenter Evaluation of Patients’ Iron Chelation with Exjade® (EPIC) study was the first to prospectively demonstrate that effective chelation can be achieved using fixed starting doses of deferasirox based on ongoing iron intake from blood transfusions, with subsequent dose titration guided by serum ferritin levels and safety markers [11]. Although deferasirox efficacy in EPIC was primarily monitored using serum ferritin, a large subset of patients was assessed by noninvasive LIC measurement using R2-magnetic resonance imaging (R2-MRI). In this subset of patients, the efficacy and safety of deferasirox in patients with lower and higher iron burdens were, therefore, assessed. This predefined analysis evaluates deferasirox for maintaining LIC in patients with baseline LIC <7 mg Fe/g dw and for reducing LIC in those with baseline LIC ≥7 mg Fe/g dw.

## Methods

The EPIC study was an international, multicenter, prospective, 1-year, open-label, phase IIIb study, for which the study design, inclusion/exclusion criteria, and dosing information have been described in full previously [11]. This paper reports the findings of a predefined liver MRI substudy of EPIC. Patients who underwent LIC assessments were recruited from the 25 centers with the appropriate apparatus and expertise to perform liver R2-MRI assessment. Eligible patients signed an additional informed consent form to participate in the MRI substudy. All study procedures were conducted in accordance with Good Clinical Practice guidelines and the Declaration of Helsinki.

### R2-MRI methodology

The magnetic resonance imagers used in the study were located at the investigators’ centers and were of a specification stipulated by Inner Vision Biometrics Pty Ltd (Claremont, WA, Australia), which conducted central analysis of the acquired data. The assessment of the R2-MRI data was conducted on-site by suitably qualified personnel. MRI systems had 1.5-T static field strengths and were capable of acquiring single spin-echo images with minimum spin-echo times of at most 6 ms. Surface chest/torso coils were used for radio frequency signal detection. MRI data analysis followed the method of St Pierre et al. [17]. Briefly, R2 values were calculated throughout a liver slice by curve fitting the equation for the biexponential decay in transverse magnetization following a single spin-echo pulse sequence to the voxel intensity data as a function of echo time. A mean R2 value was calculated for each voxel by summation of the fast and slow components of the proton transverse relaxation rate weighted by their relative population densities [17].

### Assessments

Efficacy was assessed based on changes from baseline in LIC and serum ferritin levels after 1 year of treatment with deferasirox. Patients with a baseline LIC of ≥7 mg Fe/g dw had a therapeutic goal of LIC reduction, while for patients with a baseline LIC of <7 mg Fe/g dw, the therapeutic goal was maintenance. LIC was determined at baseline, 6 months, and 12 months. Serum ferritin levels were assessed at baseline and every 4 weeks. Safety and tolerability were evaluated throughout the study by monitoring the incidence and type of adverse events (AEs) and by assessing routine laboratory parameters.

### Statistical methods

The full analysis set (FAS) population comprised all patients who successfully passed screening and were selected for participation in the R2-MRI substudy. All efficacy results are presented from the FAS population unless specified. The per protocol (PP) population comprised all patients who received the study drug and whose LIC was determined by R2-MRI at baseline and after 12 months. The safety population comprised all patients who received at least one dose of study medication and had at least one post-baseline safety assessment. Analysis sets were further divided by baseline LIC <7 and ≥7 mg Fe/g dw for the assessment of efficacy and safety. Efficacy (LIC and serum ferritin) was also assessed based on average actual deferasirox dose (<15, 15 to <25, 25 to <35, and ≥35 mg/kg/day).

Descriptive statistics were provided for LIC at baseline, 6 months, and 12 months and for the absolute change in LIC (6 months minus baseline and 12 months minus baseline). Descriptive statistics were provided for serum ferritin levels at baseline and every 4 weeks and for the absolute change in serum ferritin levels at 12 months (12 months minus baseline). For LIC change from baseline, reported *P* values are based on one-sided paired *t* tests (null hypothesis: absolute change < 3). *P* values for serum ferritin or LIC by additional subgroups are based on two-sided paired *t* tests (null hypothesis: absolute change = 0). Safety and demographic data were assessed using summary statistics. Reported *P* values for safety parameters are based on a chi-squared test. Logistic regression analyses were conducted to investigate the impact of baseline patient characteristics on the occurrence of gastrointestinal AEs in patients with baseline LIC <7 and ≥7 mg Fe/g dw. Potential confounding factors such as diagnosis, dosing, ethnicity, and history of hepatitis B and/or C were included in the analyses.

## Results

### Patient characteristics

A total of 374 patients were included in the substudy; 71 and 303 with baseline LIC <7 and ≥7 mg Fe/g dw, respectively. Baseline characteristics were generally comparable for patients with baseline LIC <7 and ≥7 mg Fe/g dw. However, the proportion of Oriental patients was lower in the <7 mg Fe/g dw cohort compared with the ≥7 mg Fe/g dw cohort (21.1 versus 48.8 %), and the proportion of patients with a history of hepatitis B and/or C was higher in the <7 mg Fe/g dw cohort than in the ≥7 mg Fe/g dw cohort (40.8 versus 21.8 %; Table 1). The majority of patients had thalassemia (81.7 and 85.5 % for the <7 and ≥7 mg Fe/g dw cohorts, respectively) and had received prior chelation therapy (84.5 and 92.4 %), most commonly with deferoxamine (DFO) monotherapy (70.4 and 63.0 %). Mean baseline LIC was 4.6 mg Fe/g dw in the baseline LIC <7 mg Fe/g dw cohort and 25.0 mg Fe/g dw in the ≥7 mg Fe/g dw cohort; median baseline serum ferritin levels were 1,479 and 4,139 ng/mL, respectively.

**Table 1:** Patient demographic and baseline characteristics

	LIC <7 mg Fe/g dw (*n* = 71)	LIC ≥7 mg Fe/g dw (*n* = 303)
Mean age ± SD, years	27.5 ± 17.8	23.1 ± 14.7
Age group, *n* (%)		
2 to <6 years	9 (12.7)	8 (2.6)
6 to <12 years	5 (7.0)	35 (11.6)
12 to 16 years	7 (9.9)	53 (17.5)
≥16 years	50 (70.4)	207 (68.3)
Female/male, *n*	35:36	154:149
Race, *n* (%)		
Caucasian	48 (67.6)	137 (45.2)
Black	3 (4.2)	7 (2.3)
Oriental	15 (21.1)	148 (48.8)
Other	5 (7.0)	11 (3.6)
Underlying disease, *n* (%)		
Thalassemia^a^	58 (81.7)	259 (85.5)
MDS	6 (8.5)	15 (5.0)
SCD	4 (5.6)	15 (5.0)
Rare anemia	2 (2.8)	6 (2.0)
Aplastic anemia	1 (1.4)	5 (1.7)
Other anemia^b^	–	3 (1.0)
History of hepatitis B and/or C, *n* (%)	29 (40.8)	66 (21.8)
Splenectomy, *n* (%)	32 (45.1)	125 (41.3)
Previous chelation therapy, *n* (%)		
DFO monotherapy	50 (70.4)	191 (63.0)
DFP monotherapy	–	3 (1.0)
DFO and DFP^c^	10 (14.1)	85 (28.1)
Other^d^	–	2 (0.7)
None	11 (15.5)	23 (7.6)
Mean number of transfusion sessions ± SD in the year prior to study entry	15.4 ± 9.3	15.8 ± 10.9
Mean volume transfused ± SD in the year prior to study entry, mL/kg	180.1 ± 231.1	158.7 ± 161.5
Mean duration of transfusions ± SD, years	19.7 ± 13.6	16.8 ± 8.8
Mean baseline LIC ± SD, mg Fe/g dw	4.6 ± 1.7	25.0 ± 10.6
Median baseline serum ferritin (range), ng/mL	1,479 (462–4,365)	4,139 (716–18,126)

*SD* standard deviation, *DFP* deferiprone

^a^β-thalassemia, hemoglobin E disease, hemoglobin E–thalassemia disease

^b^Malignant disease or congenital anemia

^c^Patients received DFP and DFO, but not necessarily in combination

^d^Other category is not mutually exclusive; patients who received DFO and/or DFP and other chelation therapies are counted under both categories

### Deferasirox dosing and iron intake

In the <7 mg Fe/g dw cohort, the majority of patients started on deferasirox 20 mg/kg/day (*n* = 67, 94.4 %); three patients received 10 mg/kg/day and one patient received 30 mg/kg/day. In the ≥7 mg Fe/g dw cohort, 171 patients (56.4 %) started on deferasirox 20 mg/kg/day and 123 (40.6 %) started on 30 mg/kg/day, 4 patients started on 10 mg/kg/day, 1 patient started on 15 mg/kg/day, and 2 patients each started on 25 or 35 mg/kg/day. Mean actual deferasirox dose in the <7 and ≥7 mg Fe/g dw cohorts was 20.7 ± 5.4 and 27.1 ± 7.1 mg/kg/day, respectively. In patients with thalassemia, the dose was 21.3 ± 5.3 and 28.3 ± 6.6 mg/kg/day in the <7 and ≥7 mg Fe/g dw cohorts, respectively.

**Fig. 1:**
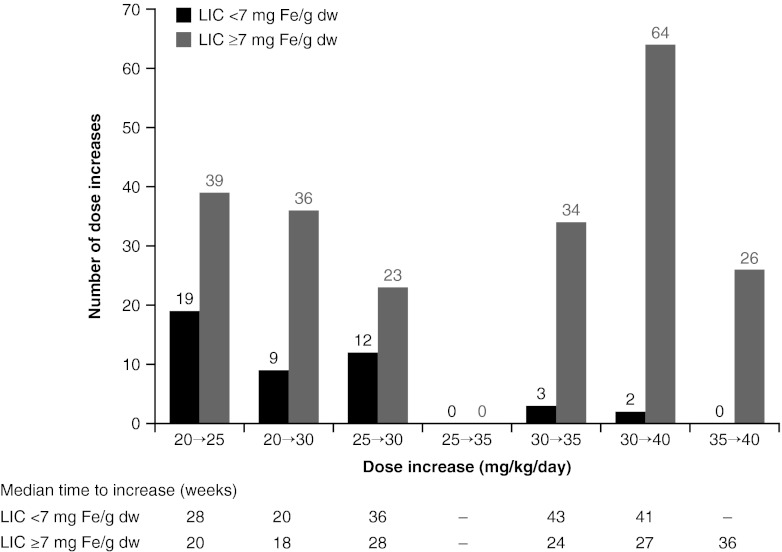
Summary of dose increases by baseline LIC category

Overall, 29 patients (40.8 %) with baseline LIC <7 mg Fe/g dw and 149 patients (49.2 %) with baseline LIC ≥7 mg Fe/g dw received dose increases during the study. The level to which doses were increased was lower in the <7 mg Fe/g dw cohort than in the ≥7 mg Fe/g dw cohort: There were only five occasions when dose was increased to >30 mg/kg/day in the <7 mg Fe/g dw cohort at any point during the study, compared with 124 occasions in the ≥7 mg Fe/g dw cohort (Fig. 1). In addition, the median time to dose increase was greater in the <7 mg Fe/g dw cohort than in the ≥7 mg Fe/g dw cohort (Fig. 1). Mean iron intake during the study was comparable in the <7 mg Fe/g dw cohort (0.38 ± 0.31 mg/kg/day) and ≥7 mg Fe/g dw cohort (0.34 ± 0.18 mg/kg/day).

### Efficacy of deferasirox in patients with LIC <7 and ≥7 mg Fe/g dw

**Fig. 2:**
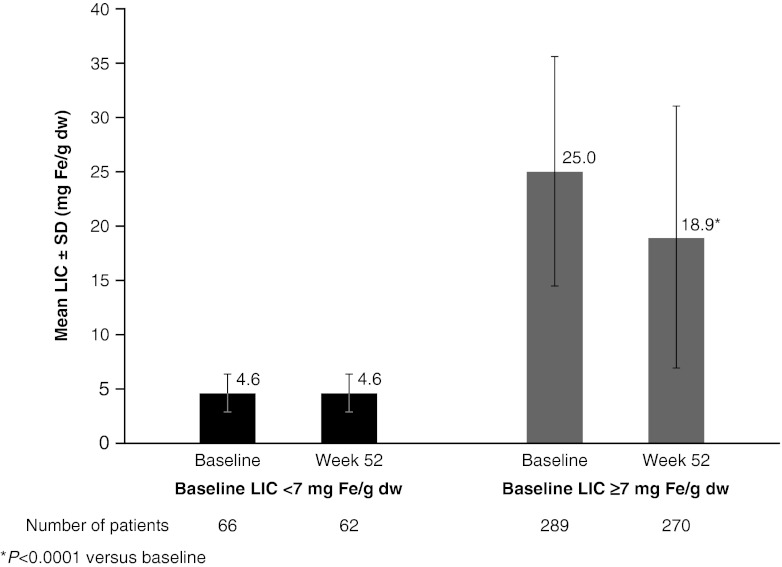
Mean LIC ± SD at baseline and after 12 months of deferasirox treatment by baseline LIC category. *P < 0.0001 versus baseline

Mean LIC was maintained over 12 months in the LIC <7 mg Fe/g dw cohort (absolute change, −0.02 ± 2.4 mg Fe/g dw; *P* = not significant), whereas in the ≥7 mg Fe/g dw cohort, mean LIC decreased significantly from 25.0 ± 10.6 mg Fe/g dw at baseline to 18.9 ± 12.0 mg Fe/g dw at 12 months (absolute change, −6.1 ± 9.1 mg Fe/g dw; *P* < 0.0001; Fig. 2). These findings were supported by results for the PP population (*n* = 332): In the LIC <7 mg Fe/g dw cohort, LIC was maintained over 12 months (4.6 ± 1.6 mg Fe/g dw at baseline and 4.7 ± 2.7 mg Fe/g dw after 12 months); in the ≥7 mg Fe/g dw cohort, mean LIC decreased from 25.0 ± 10.6 mg Fe/g dw at baseline to 19.1 ± 12.1 mg Fe/g dw after 12 months (absolute change, −6.1 ± 9.0 mg Fe/g dw). A summary of the number of patients with LIC <7, 7 to <10, and ≥10 mg Fe/g dw at baseline and after 12 months, by baseline LIC category, is shown in Table 2.

**Table 2:** Patients with LIC <7, 7 to <10, and ≥10 mg Fe/g dw at baseline and after 12 months by baseline LIC category

Baseline LIC	LIC category after 12 months^a^, mg Fe/g dw [n (%)]		
	<7	7 to <10	≥10
<7 mg Fe/g dw (*n* = 71)	63 (88.7)	4 (5.6)	4 (5.6)
7 to <10 mg Fe/g dw (*n* = 25)	15 (60.0)	10 (40.0)	–
≥10 mg Fe/g dw (*n* = 278)	31 (11.2)	36 (12.9)	211 (75.9)
Total (*n* = 374)	109 (29.1)	50 (13.4)	215 (57.5)

^a^If no value was available at 12 months, the last available post-baseline value was considered

**Fig. 3:**
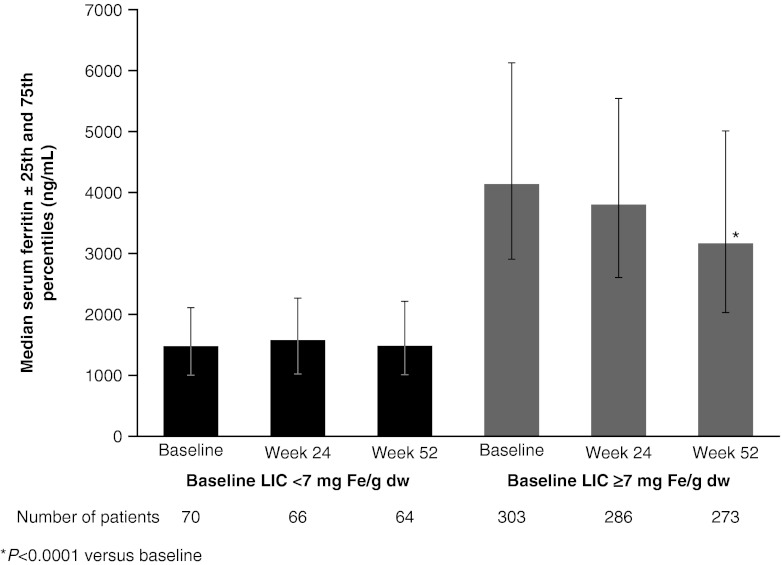
Median serum ferritin ± 25th/75th percentiles during deferasirox treatment by LIC at baseline. *P < 0.0001 versus baseline

Median serum ferritin levels were maintained at approximately baseline levels in the <7 mg Fe/g dw cohort (absolute median change, −57 ng/mL; *P* = not significant) and decreased significantly from 4,139 ng/mL at baseline to 3,176 ng/mL at 12 months in the ≥7 mg Fe/g dw cohort (absolute median change, −830 ng/mL; *P* < 0.0001; Fig. 3). The number of patients in the <7 mg Fe/g dw cohort who achieved serum ferritin levels <1,000 ng/mL by the end of the study (*n* = 16) was similar to the number at baseline (*n* = 17). In the ≥7 mg Fe/g dw cohort, the number of patients with serum ferritin levels <1,000 ng/mL increased from 3 at baseline to 16 at end of the study.

LIC and serum ferritin levels were maintained in the <7 mg Fe/g dw cohort, regardless of average actual deferasirox dose (Table 3). In the ≥7 mg Fe/g dw cohort, reductions in LIC and serum ferritin levels appeared to be dose-related (Table 3). Indeed, in contrast to the overall ≥7 mg Fe/g dw cohort, there was no significant change in serum ferritin levels in patients whose average actual deferasirox dose was <15 mg/kg/day (Table 3).

**Table 3:** Absolute changes from baseline by baseline LIC category and average actual deferasirox dose for LIC and serum ferritin

	Baseline LIC, mg Fe/g dw							
	<7 (*n* = 71)				≥7 (*n* = 303)			
	Average actual dose, mg/kg/day				Average actual dose, mg/kg/day			
	<15 (*n* = 10)	15 to <25 (*n* = 45)	25 to <35 (*n* = 16)	≥35 (*n* = 0)	<15 (*n* = 14)	15 to <25 (*n* = 100)	25 to <35 (*n* = 146)	≥35 (*n* = 43)
LIC								
Mean LIC ± SD at baseline, mg Fe/g dw	3.4 ± 1.2	4.7 ± 1.7	5.0 ± 1.4	–	16.6 ± 6.0	19.3 ± 9.2	27.5 ± 9.8	31.6 ± 9.8
Mean LIC ± SD at 12 months, mg Fe/g dw	2.6 ± 1.3	4.4 ± 2.2	6.1 ± 3.5	–	12.1 ± 9.3	14.8 ± 10.4	20.8 ± 12.0	23.9 ± 12.6
Mean change in LIC ± SD, mg Fe/g dw	−0.4 ± 1.1	−0.2 ± 2.1	0.7 ± 3.3	–	−4.6 ± 4.7	−4.2 ± 7.0	−6.9 ± 9.6	−7.3 ± 10.7
*P* value^a^	NS	NS	NS	–	0.0442	<0.0001	<0.0001	<0.0001
Serum ferritin								
Median serum ferritin at baseline, ng/mL	878	1,527	1,882	–	2,464	3,151	4,555	6,230
Median serum ferritin at 12 months, ng/mL	966	1,553	1,593	–	2,799	2,395	3,693	5,141
Median change in serum ferritin, ng/mL	−28	−62	−16	–	154	−434	−1,003	−1,496
*P* value^a^	NS	NS	NS	–	NS	0.0121	<0.0001	0.0004

*NS* not significant, *SD* standard deviation

^a^Paired two-sided *t* test based on mean change from baseline

### Discontinuations and safety

Overall, 63 out of 71 (88.7 %) and 274 out of 303 (90.4 %) patients in the <7 and ≥7 mg Fe/g dw cohorts completed the study, respectively; 54 and 241 of these were patients with thalassemia. Reasons for discontinuation in the <7 and ≥7 mg Fe/g dw cohorts were, respectively, AEs (*n* = 6, 8.5 % and *n* = 11, 3.6 %), consent withdrawal (0 % and *n* = 4, 1.3 %), unsatisfactory therapeutic effect (*n* = 1, 1.4 % and *n* = 1, 0.3 %), abnormal laboratory value (*n* = 1, 1.4 % and *n* = 1, 0.3 %), protocol violation (0 % and *n* = 4, 1.3 %), no longer required study drug (0 % and *n* = 2, 0.7 %), administrative problems (0 % and *n* = 2, 0.7 %), loss to follow-up (0 % and *n* = 1, 0.3 %), and death (0 % and *n* = 3, 1 % [pneumonia, *n* = 1; sepsis, *n* = 2]).

The AEs leading to discontinuation (regardless of relationship to deferasirox) in the <7 mg Fe/g dw cohort were diarrhea (*n* = 2), eyelid edema (*n* = 1), generalized edema (*n* = 1), cholelithiasis (*n* = 1), and disease progression (*n* = 1). AEs leading to discontinuation in the ≥7 mg Fe/g dw cohort were sepsis (*n* = 3), vomiting (*n* = 2), cardiomyopathy, abdominal pain, constipation, diarrhea, nausea, adverse drug reaction, pneumonia, increased blood creatinine, hemosiderosis, fistula, muscle weakness, dizziness, nephritis, acute renal failure, renal tubular disorder, angioedema, swelling face, and urticaria (all *n* = 1; note that patients could have multiple AEs reported as reason for discontinuation). The most common investigator-assessed drug-related AEs observed in each LIC cohort are shown in Table 4.

**Table 4:** Most common investigator-assessed drug-related AEs (≥5 % in either group overall) by baseline LIC category and severity

AE, *n* (%)	Baseline LIC, mg Fe/g dw							
	<7 (*n* = 71)				≥7 (*n* = 303)			
	Severity				Severity			
	Mild	Mod	Severe	Total	Mild	Mod	Severe	Total
Diarrhea	15 (21.1)	2 (2.8)	1 (1.4)	18 (25.4)	26 (8.6)	5 (1.7)	0	31 (10.2)
Abdominal pain	7 (9.9)	2 (2.8)	0	9 (12.7)	9 (3.0)	3 (1.0)	0	12 (4.0)
Upper abdominal pain	6 (8.5)	1 (1.4)	0	7 (9.9)	12 (4.0)	1 (0.3)	0	13 (4.3)
Constipation	6 (8.5)	1 (1.4)	0	7 (9.9)	9 (3.0)	0	0	9 (3.0)
Nausea	4 (5.6)	2 (2.8)	0	6 (8.5)	11 (3.6)	5 (1.7)	0	16 (5.3)
Abdominal distension	5 (7.0)	0	0	5 (7.0)	7 (2.3)	1 (0.3)	0	8 (2.6)
Rash	4 (5.6)	1 (1.4)	0	5 (7.0)	21 (6.9)	14 (4.6)	4 (1.3)	39 (12.9)
Abnormal LF test	3 (4.2)	1 (1.4)	0	4 (5.6)	2 (0.7)	1 (0.3)	0	3 (1.0)
Increased blood creatinine	0	0	0	0	29 (9.6)	9 (3.0)	0	38 (12.5)

*LF* liver function, *mod* moderate

In the <7 mg Fe/g dw cohort, there was one case of investigator-assessed drug-related severe diarrhea in a patient with MDS. In the ≥7 mg Fe/g dw cohort, there were four cases of investigator-assessed drug-related severe rash; three of these occurred in patients with thalassemia, the fourth in a patient with MDS. The most frequently reported drug-related AEs were gastrointestinal (Table 4). These occurred more frequently in the <7 mg Fe/g dw cohort than the ≥7 mg Fe/g dw cohort (39.4 versus 20.8 %; *P* = 0.001). Rates of discontinuation as a result of drug-related gastrointestinal AEs were low: Two patients in the LIC <7 mg Fe/g dw cohort discontinued as a result of diarrhea and three patients in the ≥7 mg Fe/g dw cohort discontinued as a result of constipation, diarrhea, nausea, and vomiting.

Multivariate analysis was conducted in order to determine whether any baseline patient characteristics influenced the observed difference in the occurrence of drug-related gastrointestinal AEs between the <7 and ≥7 mg Fe/g dw cohorts. This analysis revealed that patients with LIC <7 mg Fe/g dw have a higher risk of gastrointestinal AEs, which was not confounded by diagnosis, dosing, ethnicity, or history of hepatitis B and/or C. This analysis also demonstrated a mild interactive effect between LIC <7 mg Fe/g dw and history of hepatitis B and/or C, resulting in a higher incidence of gastrointestinal AEs in thalassemia patients with both LIC <7 mg Fe/g dw and a history of hepatitis B and/or C compared with patients with LIC <7 mg Fe/g dw and no history of hepatitis B and/or C (46.2 versus 28.1 %, respectively). However, this did not reach statistical significance.

When determining whether ethnicity influenced the incidence of gastrointestinal AEs, the analysis showed that non-Oriental patients had a higher risk of gastrointestinal AEs, which was not confounded by diagnosis, baseline LIC category, or history of hepatitis B and/or C. Eighteen (11.0 %) Oriental patients were reported to have a gastrointestinal AE compared with 6 (60.0 %), 55 (30.1 %), and 12 (75.0 %) patients of Black, Caucasian, and other ethnicity, respectively.

Serious AEs were noted in 6 patients (8.5 %) in the <7 mg Fe/g dw cohort and 38 patients (12.5 %) in the ≥7 mg Fe/g dw cohort. None of the serious AEs occurring in the <7 mg Fe/g dw cohort were considered to be drug-related by the investigator. In the ≥7 mg Fe/g dw cohort, three patients (all with β-thalassemia) experienced serious AEs considered to be drug-related; one patient experienced renal tubular disorder of moderate intensity and the other two patients experienced serious AEs of severe intensity (angioedema and nephritis/acute renal failure). Three patients (all in the ≥7 mg Fe/g dw cohort) died during the study; one patient with underlying aplastic anemia died as a result of pneumonia and two patients (one with underlying malignant disease and one with underlying thalassemia) died as a result of sepsis. No cause of death was considered to be drug-related.

Overall, 21 (29.6 %) patients in the <7 mg Fe/g dw cohort and 105 (34.7 %) patients in the ≥7 mg Fe/g dw cohort had two or more consecutive increases in serum creatinine >33 % above baseline; all patients had low or normal levels at baseline. The proportion of patients with two or more consecutive increases in serum creatinine levels >33 % above baseline and above the upper limit of normal (ULN) was similar in the <7 mg Fe/g dw cohort (*n* = 5, 7.0 %; thalassemia, *n* = 4; MDS, *n* = 1) and ≥7 mg Fe/g dw cohort (*n* = 16, 5.3 %; thalassemia, *n* = 8; MDS, *n* = 5; SCD, *n* = 1; malignant disease, *n* = 1; rare anemia, *n* = 1). Five of these patients subsequently received specific dose reductions for a laboratory test abnormality. When investigator-reported AEs are considered (Table 4), increased blood creatinine was not reported more frequently in patients with baseline LIC <7 mg Fe/g dw compared with ≥7 mg Fe/g dw.

Three patients had two consecutive alanine aminotransferase (ALT) values >10 times ULN during deferasirox treatment: one (1.4 %) in the <7 mg Fe/g dw cohort (thalassemia and hepatitis B/C-positive) and two (0.7 %) in the ≥7 mg Fe/g dw cohort (thalassemia and hepatitis B/C-positive, *n* = 1; thalassemia and hepatitis B/C-negative, *n* = 1). Both hepatitis B/C-positive patients received dose reductions/interruptions as a result of laboratory test abnormalities, with ALT levels returning to within the normal range.

## Discussion

With widening therapeutic and investigative/monitoring options in iron chelation therapy, an emerging theme includes the benefits and safety of achieving target LIC <7 mg Fe/g dw. In this 1-year substudy of the EPIC trial, LIC and serum ferritin levels were successfully maintained in patients with baseline LIC <7 mg Fe/g dw, while those with baseline LIC ≥7 mg Fe/g dw achieved significant reductions, with 60.0 % of patients with baseline LIC 7 to <10 mg Fe/g dw shifting to LIC <7 mg Fe/g dw after 12 months; it is probable that treatment for longer than 1 year would be required for many patients with higher baseline LICs to achieve their therapeutic goal. Such effects on iron balance appeared to be dose-related, since maintenance of LIC and serum ferritin levels in patients with baseline LIC <7 mg Fe/g dw was achieved at a lower mean dose of 20.7 mg/kg/day, whereas in those with baseline LIC ≥7 mg Fe/g dw, significant reductions in LIC and serum ferritin were achieved at a mean dose of 27.1 mg/kg/day. This supports the findings from a previous trial, which suggested that a deferasirox dose of 20 mg/kg/day is a suitable maintenance dose and doses of 30 mg/kg/day are required to achieve negative iron balance in regularly transfused β-thalassemia patients [18]. Moreover, subanalyses in the present study based on average actual deferasirox dose indicated dose-related reductions in LIC and serum ferritin levels in the ≥7 mg Fe/g dw cohort. The median time to a dose increase in this 52-week study was relatively long, ranging from 18 to 43 weeks. As some patients require higher doses to achieve their therapeutic goal, it might reasonably be hypothesized that a greater proportion of patients would have achieved their therapeutic goal if dose increases had been carried out more promptly. More rapid dose escalations are particularly important in patients requiring a reduction in iron burden, such as those with LIC ≥7 mg Fe/g dw.

The data presented here from the EPIC study build on data already shown for a smaller number of patients with LIC <7 and ≥7 mg Fe/g dw in the ESCALATOR trial [16]. In ESCALATOR, patients with β-thalassemia who were previously unsuccessfully chelated with DFO and/or deferiprone (DFP) also achieved their therapeutic goal of LIC reduction when treated with deferasirox despite high iron burden at baseline (90 % had LIC ≥7 mg Fe/g dw) [16]. In our subset of patients, the proportion with LIC ≥7 mg Fe/g dw was slightly lower (81 %) and that with LIC <7 mg Fe/g dw was higher (19 %) than in the ESCALATOR study, allowing a more detailed comparison between the low and high iron burden groups. In addition, the present study expands these findings to patients with other transfusion-dependent anemias, including MDS, SCD, and other rare anemias that were also included in this MRI substudy.

Although the safety profile of deferasirox has been investigated in many trials, there are limited data evaluating deferasirox doses of 20 mg/kg/day in patients with a lower iron burden. The ESCALATOR trial found no clear trends in the type or frequency of drug-related AEs between the LIC <7 and ≥7 mg Fe/g dw cohorts [16]. In addition, a small study (*n* = 19) by Grosse et al. showed no safety concerns at a median deferasirox dose of 19 mg/kg/day in patients with LIC <6 mg Fe/g dw [15]. Safety evaluation of larger patient numbers, as seen here, was required to corroborate these findings. In this study, deferasirox treatment had a clinically manageable safety profile in patients with baseline LIC <7 and ≥7 mg Fe/g dw, thus confirming the tolerability profile of deferasirox 20 mg/kg/day in patients with lower iron burdens. The discontinuation rate as a result of AEs was low in both groups and only four patients overall withdrew consent. Interestingly, fewer patients in the <7 mg Fe/g dw cohort experienced drug-related rash and increased blood creatinine noted as investigator-assessed AEs. Laboratory data confirming two or more consecutive increases in serum creatinine >33 % above baseline over 1 year were consistent with previous studies [18–20]. Importantly, this study did not show an increased risk of renal toxicity at LIC <7 mg Fe/g dw compared with those patients treated at higher LIC values.

Patients in the <7 mg Fe/g dw cohort experienced more gastrointestinal AEs than those in the ≥7 mg Fe/g dw cohort. The multivariate analysis showed that the higher risk of gastrointestinal AEs in patients within the LIC <7 mg Fe/g dw cohort occurred irrespective of diagnosis, dosing, ethnicity, and history of hepatitis B and/or C. This might suggest that these generally mild-to-moderate gastrointestinal effects are exacerbated by iron deprivation in the gut as a result of chelation therapy, but this requires confirmation. There was also an indication that patients with low baseline LIC who have a history of hepatitis B and/or C are at a higher risk of gastrointestinal AEs compared with those with low baseline LIC without a history of hepatitis B and/or C, although this interaction was not statistically significant and, therefore, requires confirmation through additional studies.

Non-Oriental patients had a higher risk of gastrointestinal AEs irrespective of diagnosis, baseline LIC category, and history of hepatitis B and/or C. Differences in safety profile between ethnic backgrounds in the EPIC study have also been recently highlighted elsewhere, whereby patients from the Asia-Pacific region (89.0 % Oriental background) reported a higher incidence of skin rash compared with those in the rest of the world [21]. It is, therefore, possible that pharmacogenetics may be important when managing patients treated with deferasirox; however, further investigations are warranted.

In conclusion, this study confirms that deferasirox doses of 20 mg/kg/day over 1 year enables patients to maintain LIC <7 mg Fe/g dw and that deferasirox doses of approximately 30 mg/kg/day are required to achieve a significant reduction in LIC in patients with baseline LIC ≥7 mg Fe/g dw. There was a clinically manageable safety profile in both LIC cohorts. The increased incidence of mild-to-moderate gastrointestinal AEs in patients with LIC <7 mg Fe/g dw requires confirmation in other patient groups to provide further insight into possible mechanisms for this observation.
